# Respiratory Infections Precede Adult-Onset Asthma

**DOI:** 10.1371/journal.pone.0027912

**Published:** 2011-12-21

**Authors:** Aino Rantala, Jouni J. K. Jaakkola, Maritta S. Jaakkola

**Affiliations:** 1 Center for Environmental and Respiratory Health Research, Institute of Health Sciences, University of Oulu, Oulu, Finland; 2 Respiratory Medicine Unit, Center for Environmental and Respiratory Health Research, Institute of Clinical Medicine, University of Oulu and Oulu University Hospital, Oulu, Finland; University of Tübingen, Germany

## Abstract

**Background:**

Respiratory infections in early life are associated with an increased risk of developing asthma but there is little evidence on the role of infections for onset of asthma in adults. The objective of this study was to assess the relation of the occurrence of respiratory infections in the past 12 months to adult-onset asthma in a population-based incident case-control study of adults 21–63 years of age.

**Methods/PrincipalFindings:**

We recruited all new clinically diagnosed cases of asthma (n = 521) during a 2.5-year study period and randomly selected controls (n = 932) in a geographically defined area in South Finland. Information on respiratory infections was collected by a self-administered questionnaire. The diagnosis of asthma was based on symptoms and reversible airflow obstruction in lung function measurements. The risk of asthma onset was strongly increased in subjects who had experienced in the preceding 12 months lower respiratory tract infections (including acute bronchitis and pneumonia) with an adjusted odds ratio (OR) 7.18 (95% confidence interval [CI] 5.16–9.99), or upper respiratory tract infections (including common cold, sinusitis, tonsillitis, and otitis media) with an adjusted OR 2.26 (95% CI 1.72–2.97). Individuals with personal atopy and/or parental atopy were more susceptible to the effects of respiratory infections on asthma onset than non-atopic persons.

**Conclusions/Significance:**

This study provides new evidence that recently experienced respiratory infections are a strong determinant for adult-onset asthma. Reducing such infections might prevent onset of asthma in adulthood, especially in individuals with atopy or hereditary propensity to it.

## Introduction

Asthma is a chronic inflammatory disease of the airways characterized by reversible airflow obstruction [1]. It is among the most common chronic diseases in working-age adults with an average prevalence of 7.5% in Finland [2] and 7.3% in United States [3].

Respiratory infections may play a role in the etiology of asthma. Children who experience viral respiratory infections in early life are more likely to develop asthma later in childhood [4]–[7]. However, there is little data on the role of infections for onset of asthma in adults. Serologic evidence of chronic *Chlamydia pneumoniae* infection has been linked to adult asthma [8], [9], but acute respiratory infections as determinants of adult-onset asthma have not been studied. However, there are plausible mechanisms by which infectious agents could contribute to induction of asthma [10], [11]. Respiratory infections cause airway epithelial damage and airway inflammation and initiate immune responses that further enhance airway inflammation.

The objective of this study was to assess the relation between occurrence of respiratory infections in the past 12 months and onset of adult asthma in a population-based incident case-control study of a working age population. In addition, potential interaction between atopy and infections for the adult-onset asthma was evaluated.

## Methods

### Study design

This study was a population-based incident case-control study. The source population consisted of adults 21 to 63 years of age living in the Pirkanmaa Hospital District, a geographically defined administrative area in South Finland (population 440 913 in 1997). We recruited all the new cases of asthma during a 2.5-year study period and randomly selected controls from the source population. All study subjects signed an informed consent form. The study was approved by the ethics committees of the Finnish Institute of Occupational Health and the Tampere University Hospital.

### Definition and selection of cases

We recruited systematically all the new cases of asthma 1997–2000 at all health care facilities diagnosing asthma in the Pirkanmaa Hospital district, including the Department of Pulmonary Medicine at the Tampere University Hospital, offices of the private-practicing pulmonary physicians in the region as well as public health care centers. As an additional route of case recruitment, the National Social Insurance Institution of Finland invited all patients, who had received reimbursement rights for asthma medication during our study period, but had not yet participated. The diagnostic criteria for asthma are given in Table 1 [2], [12]–[19].

**Table 1:** Diagnostic criteria for asthma.

**1. Occurrence of at least one asthma-like symptom: prolonged cough, wheezing, attacks of or exercise-induced dyspnea, or nocturnal cough or wheezing**
*and*
**2. Demonstration of significant reversibility in airways obstruction in lung function investigations:**
Significant improvement in response to short-acting bronchodilating medication in a bronchodilator test after baseline spirometry or at the end of methacholine challenge. The criteria for significant changes were [12]: FEV_1_≥15%, FVC≥15%, PEF≥23%
*and/or*
≥20% daily variation^a^ and/or ≥15% improvement^a^ in response to short-acting bronchodilating medication during at least two days in a 2-week diurnal PEF monitoring
*and/or*
Significant improvement in spirometric lung function (for % criteria see above)
*and/or*
≥20% improvement in the average PEF level in response to a 2-week oral steroid treatment

FEV_1_ = forced expiratory volume in one second; FVC = forced vital capacity; PEF = peak expiratory flow.

a Calculated according to the standard practice of the Tampere University Hospital: maximum daily variation = (highest PEF value during the day – lowest PEF value during the day)/highest PEF value during the day; bronchodilator response = (highest PEF value after bronchodilating medication – highest PEF value before medication)/highest PEF value before medication.

The medical records of all cases were checked, and only those with no previously diagnosed asthma or long-term use of any asthma medication were included in the study to ensure that the cases had asthma diagnosed for the first time at recruitment. At the Tampere University Hospital, cases were recruited even before the diagnosis (at their first visit due to suspected asthma) and the diagnosis was then verified in clinical examinations. A total of 521 cases participated (response rate 86%).

### Selection of controls

The controls were randomly drawn from the source population using the national population registry, which has full coverage of the population. The general eligibility criteria were applied for controls (living in Pirkanmaa, age 21–63 years). Recruitment of controls took place by a letter at six-month intervals throughout the study period. A total of 1016 subjects participated in the study (response rate 80%). Previous or current asthma was reported by 76 (7.5%), six persons were older than 64 years and two returned incomplete questionnaire. After excluding these persons, our study population included 932 controls.

## Methods

### Questionnaire

The study subjects answered a self-administered questionnaire [2], [13], [15]–[17], [19]–[21] that included six sections: 1) personal characteristics, 2) health information and family history of atopic diseases, 3) active smoking and secondhand tobacco smoke (SHS) exposure, 4) occupation and work environment, 5) home environment, and 6) dietary questions.

### Lung function measurements

The same diagnostic protocol was applied for all patients with suspicion of asthma (Table 1). The spirometry and bronchodilation test were recorded with a pneumotachograph spirometer using disposable flow transducer (Medikro 905, Medikro, Kuopio, Finland) according to the standards of the American Thoracic Society [22]. The details of this and other lung function measurements are given elsewhere [2], [12]. Presence of obstruction was judged using the reference values derived from the Finnish population [23].

### Assessment of the occurrence of respiratory infections

Occurrence of respiratory infections was assessed based on the following question: *How often did you experience the following infections during the past 12 months and the past 3 months?* The list of infections included common cold, tonsillitis, sinusitis, otitis media, acute bronchitis, and pneumonia. The infections were categorized according to at least one infection (≥1) versus no infection (the reference), except for common colds according to at least two infections (≥2) versus zero to one infection (the reference). Respiratory infections were classified as lower respiratory tract infections (LRTI; including acute bronchitis, and pneumonia) and upper respiratory tract infections (URTI; including common cold, tonsillitis, sinusitis, and otitis media).

### Statistical methods

We used exposure odds ratio (OR) with 95% confidence interval (CI) derived from logistic regression models to quantify the relations between occurrence of infections and the risk of asthma. Sex, age, education (as an indicator of socio-economic status), personal smoking, mold problems in the home or at work, exposure to SHS in the home or at work, and any history of pets in the home were adjusted for as covariates.

We also studied independent and joint effects of both personal allergic disease and parental allergic disease and LRTIs by comparing the risk of asthma in four exposure categories: (1) no personal/parental allergy and no LRTIs during the past 12 months (the reference category); (2) personal/parental allergy and no LRTIs; (3) no personal/parental allergy but experienced LRTIs in past 12 months; and (4) personal/parental allergy and LRTIs. ORs were calculated by contrasting each of the three exposure categories with the reference category. Estimates for the independent effects and joint effect were derived from the same logistic regression model, adjusting for the covariates. Joint effects were assessed on additive scale from these models.

Personal allergy was defined as a history of allergic rhinitis or dermatitis diagnosed by a doctor. Parental allergy was defined as a history of maternal or paternal asthma, hay fever, allergic eczema, or allergic conjunctivitis.

Statistical analyses were performed using SAS statistical package (SAS Institute Inc., Cary, NC, USA). A graphic was prepared using the free software package R (R Development Core Team, 2006).

## Results

### Respiratory infections and the risk of asthma

**Figure 1:**
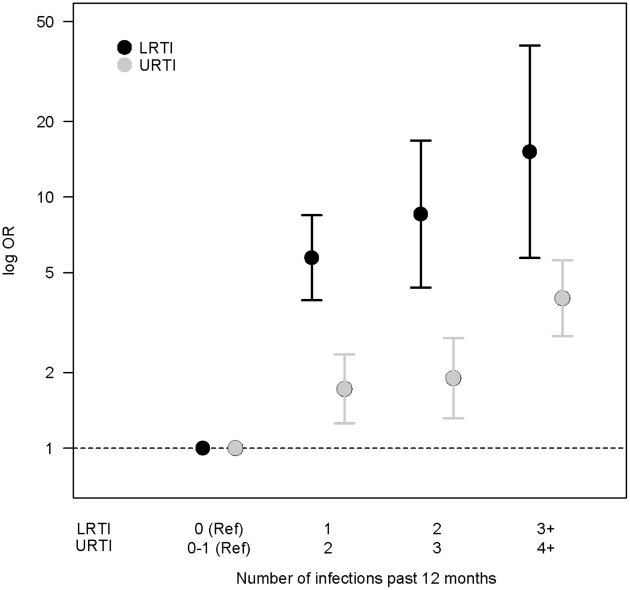
Exposure-response patterns for LRTIs and URTIs in the past 12 months. X axis shows increasing number of infections (reference category: no infections for LRTIs and zero to one infections for URTIs) and Y axis shows logarithmic ORs with 95% confidence intervals for asthma onset.

Characteristics of the cases and controls are presented in Table 2. Table 3 shows that cases of adult-onset asthma had experienced more commonly both LRTIs and URTIs in the past 12 months compared to controls (35.8% vs. 7.3% and 33.8% vs. 17.8%, respectively). The risk of asthma onset was significantly increased in relation to LRTIs (adjusted OR 7.18; 95% CI: 5.16–9.99) and URTIs (adjusted OR 2.26; 95% CI: 1.72–2.97) in the past 12 months. Significant increases of asthma risk were also detected when infections were considered separately, i.e. acute bronchitis, pneumonia, common cold, sinusitis, and otitis media. Only the adjusted OR for tonsillitis did not reach significance. We evaluated exposure-response patterns by estimating ORs of asthma according to increasing number of infections compared to no (LRTIs) or zero to one infections (URTIs) in the past 12 months. Figure 1 illustrates a clear exposure-response relation for LRTIs (trend test: Wald Chi-square 146.8, P<0.0001) and a suggestive exposure-relation for URTIs (trend test: Wald Chi-square 3.59, P = 0.0581).

**Table 2:** Characteristics of the study population.

Characteristic		Cases	Controls
		N (%)	N (%)
Total		521	932
Sex	Male	175 (33.6)	346 (66.4)
	Female	438 (47.0)	494 (53.0)
Age (years)	21–29	108 (20.7)	141 (15.1)
	30–39	107 (20.5)	224 (24.0)
	40–49	125 (24.0)	254 (27.3)
	50–59	140 (26.9)	240 (25.8)
	60–64	41 (7.9)	73 (7.8)
Education^a^	No vocational schooling	107 (20.6)	154 (16.6)
	Vocational course	89 (17.2)	104 (11.2)
	Vocational institution	149 (28.7)	271 (29.2)
	College-level education	113 (21.8)	261 (28.1)
	University or corresponding	61 (11.8)	138 (14.9)
Smoking^b^	No	239 (46.1)	487 (52.4)
	Ex	133 (25.7)	203 (21.8)
	Current	146 (28.2)	240 (25.8)
Pets	Never	155 (29.8)	316 (33.9)
	Past 12 months	10 (1.9)	31 (3.3)
	>1 year ago	356 (68.3)	585 (62.8)
Personal allergy^c^	Never	218 (41.8)	555 (59.6)
	Current or in the past	303 (58.2)	377 (40.6)
Parental atopic diseases		186 (35.7)	204 (21.9)
SHS in the workplace		89 (17.1)	130 (13.9)
SHS in the home		30 (5.9)	52 (5.6)
Visible mold or mold odor in the home		70 (13.4)	118 (12.7)
Visible mold or mold odor in the workplace		70 (13.4)	99 (10.6)

SHS = secondhand tobacco smoke.

a Information on education was missing for 6 subjects.

b Information on smoking was missing for 5 subjects.

c Rhinitis or dermatitis.

**Table 3:** Occurrence of respiratory infections in the past 12 months and in the past 3 months and the risk of adult-onset asthma.

Infection (≥1 infections)	Cases	Controls		
	N (%)	N (%)	Crude OR (95% CI)	Adjusted OR (95% CI)^a^
Total^b^	521	932		
**During the past 12 months**				
Lower respiratory tract infections	182 (35.8)	65 (7.3)	7.10 (5.20–9.68)	7.18 (5.16–9.99)
*Acute bronchitis*	174 (34.6)	64 (7.2)	6.85 (5.01–9.37)	7.03 (5.04–9.81)
*Pneumonia*	20 (3.9)	5 (0.6)	7.27 (2.71–19.47)	6.46 (2.37–17.63)
Upper respiratory tract infections	172 (33.8)	159 (17.8)	2.36 (1.84–3.03)	2.26 (1.72–2.97)
*Common cold* c	258 (50.9)	297 (33.2)	2.08(1.67–2.60)	2.11 (1.65–2.69)
*Tonsillitis*	36 (7.1)	40 (4.5)	1.63 (1.02–2.59)	1.61 (0.99–2.62)
*Sinusitis*	140 (27.5)	109 (12.2)	2.73 (2.07–3.61)	2.64 (1.95–3.58)
*Otitis media*	39 (7.7)	34 (3.8)	2.10 (1.31–3.37)	2.09 (1.27–3.43)
**During the past 3 months**				
Lower respiratory tract infections	48 (9.4)	23 (2.6)	3.94 (2.37–6.56)	4.03 (2.35–6.92)
*Acute bronchitis*	47 (9.2)	23 (2.6)	3.85 (2.31–6.42)	3.95 (2.29–6.79)
*Pneumonia*	2 (0.4)	0 (0)	-	-
Upper respiratory tract infections	226 (44.4)	311 (34.8)	1.50 (1.20–1.87)	1.51 (1.19–1.92)
*Common cold*	197 (38.7)	289 (32.3)	1.32 (1.05–1.66)	1.32 (1.03–1.68)
*Tonsillitis*	9 (1.8)	16 (1.8)	0.99 (0.43–2.25)	1.03 (0.44–2.44)
*Sinusitis*	62 (12.2)	40 (4.5)	2.96 (1.96–4.48)	3.08 (1.98–4.78)
*Otitis media*	13 (2.6)	10 (1.1)	2.32 (1.01–5.32)	2.52 (1.01–6.29)

a Adjusted for sex, age, education, smoking, SHS exposure (work/home), pets, and exposure to mold (work/home).

b Total number of cases and controls, information on infections was missing for 50 subjects.

c ≥2 infections.

Both LRTIs and URTIs were also more common in cases than controls when experienced during the past three months (9.4% vs. 2.6% and 44.4% vs. 34.8%, respectively). Adjusted ORs were 4.03 (95% CI: 2.35–6.92) for LRTIs and 1.51 (95% CI: 1.19–1.92) for URTIs. When analyzed according to each infection separately, all other respiratory infections in the past 3 months significantly increased the risk of adult-onset asthma, expect tonsillitis (altogether only 25 episodes of tonsillitis were reported). An OR for pneumonia could not be calculated, because only two pneumonias were reported in the last three months, but both of these were among asthma cases (Table 3).

### Joint effect of LRTIs and allergic diseases

We analyzed the joint effect of LRTIs experienced during the past 12 months and personal allergic diseases (currently or in the past) on the risk of asthma (Table 4). Subjects with allergic disease but no LRTIs during the past 12 months had an increased risk for asthma with an adjusted OR of 1.98 (95% CI: 1.49–2.62), corresponding to a 98% excess risk compared to nonallergic subjects with no LRTIs (the reference category). The effect of LRTIs on adult-onset asthma in nonallergic subjects was also significant with an OR of 8.49 (95% CI: 5.16–13.97), which corresponds to a 749% excess risk. In subjects with both allergic disease and LRTIs the adjusted OR for asthma was 11.38 (95% CI: 7.25–17.85) corresponding to an excess risk of 1038%. This suggests a slight synergistic effect of atopy and LRTIs on the additive scale.

**Table 4:** Joint effect of LRTIs (≥1 infections) experienced during the past 12 months and personal allergic diseases (currently or in the past) on the risk of asthma.

Allergy	LRTI	Crude OR (95% CI)	Adjusted OR (95% CI)^a^	Excess risk (%)
No	No	1	1	
Yes	No	1.93 (1.49–2.50)	1.98 (1.49–2.62)	98
No	Yes	7.65 (4.81–12.16)	8.49 (5.16–13.97)	749
Yes	Yes	11.69 (7.63–17.90)	11.38 (7.25–17.85)	1038

a Adjusted for sex, age, education, smoking, SHS exposure (work/home), pets, and exposure to mold (work/home).

In addition, we analyzed the joint effect of LRTIs and a history of parental allergic diseases on the risk of asthma (Table 5). Those subjects with parental allergic disease but no LRTIs during the past 12 months had a 91% excess risk for asthma (adjusted OR 1.91, 95% CI 1.42–2.58) compared to those with no parental allergy and no LRTIs (the reference category). The excess risk for asthma in subjects with LRTIs but no parental allergy was 697% (adjusted OR 7.97, 95% CI 5.31–11.95). The effect of both parental allergy and LRTIs suggested a slight synergistic effect on the additive scale with an OR of 9.97 (95% CI: 5.80–17.13), which corresponds to an 897% excess risk.

**Table 5:** Joint effect of LRTIs (≥1 infections) experienced during the past 12 months and parental allergic diseases on the risk of asthma.

Parental allergy	LRTI	Crude OR (95% CI)	Adjusted OR (95% CI)^a^	Excess risk (%)
No	No	1	1	
Yes	No	1.97 (1.49–2.61)	1.91 (1.42–2.58)	91
No	Yes	7.94 (5.43–11.63)	7.97 (5.31–11.95)	697
Yes	Yes	9.93 (5.94–16.59)	9.97 (5.80–17.13)	897

a Adjusted for sex, age, education, smoking, SHS exposure (work/home), pets, and exposure to mold (work/home).

## Discussion

### Main findings

The results of this study show that occurrence of respiratory tract infections in the preceding 12 months is a strong determinant for the onset of adult asthma. This large population-based incident case-control study that included a follow-up of 581,000 person-years showed that both upper and lower respiratory tract infections 12 months prior to the asthma-onset are significant determinants of asthma in a working age population. Recently experienced LRTIs, including acute bronchitis and pneumonia, were associated with an especially high OR for asthma. There was evidence for an exposure-response relation: the asthma risk increased with an increasing number of both LRTIs and URTIs. A significant increase in asthma-onset risk was seen in association to all types of respiratory infections. The only exception to this consistent pattern was occurrence of tonsillitis.

There are plausible biological pathways that could underlie these observations. Besides microbial destruction, immune system against respiratory infectious agents may lead to chronic type of inflammation of the airways and tissue damage. Recently, Kim et al. [10] introduced a mouse model that provided new evidence of how chronic lung disease may develop after infection with a respiratory virus. They infected mouse with a parainfluenza virus Sendai, which is similar to common human respiratory viruses. An acute immune defense against infection was followed by a chronic inflammatory response, where activation of natural killer T cells and macrophages in the lung promoted persistent production of IL-13. Persistent IL-13 then caused chronic mucous cell metaplasia and airway hyperreactivity. They also showed that similar innate immune axis operated in humans with asthma and chronic obstructive pulmonary disease. Thus, the innate immune system may have a pathogenic role in asthma onset after respiratory infection. So respiratory infections could act as the primary inducers of inflammation leading to the development of asthma, or they may act as triggers of onset in subjects already experiencing inflammation due to other factors, such as environmental exposures.

The risk of asthma related to both LRTIs and URTIs was stronger when assessing occurrence of infections in the past 12 months as compared to infections in the past 3 months. This could be explained partly by the smaller number of infections experienced in the past 3 months, so the estimates for the latter may be less stable. On the other hand, the stronger effect of infections in the past year could be compatible with a causal influence, as a chronic inflammatory response induced by an infectious agent could be expected to need more than three months to become severe enough to cause a clinical disease with symptoms and measurable changes in lung function. Thus, the past 12 months captures better the relevant time period for induction of asthma than the past 3 months.

We used a history of parental allergic diseases as a measure of hereditary propensity to allergy. Consistently with previous findings, parental allergy was an independent determinant of asthma onset [13]. In addition, the results showed that a combination of parental allergy and LRTIs had a synergistic effect on asthma onset on the additive scale. Interestingly, the occurrence of LRTIs was a stronger independent determinant for onset of asthma than the history of parental allergy alone. Also personal allergy was related to asthma, and a combination of personal allergy and LRTIs had a synergistic effect on asthma-onset. These findings suggest that subjects with personal allergy or hereditary propensity to allergy are more susceptible to the effects of LRTIs on asthma risk. Similar results have been reported in children [24]–[26]. In those studies the highest risk of asthma was seen in children who were both atopic and experienced early LRTIs. Atopy and LRTIs seem to be independent determinants of asthma onset suggesting different inflammatory pathways. However, an interaction of these disparate inflammatory stimuli has a synergistic effect on asthma risk.

Overall, our results suggest that reducing occurrence of respiratory infections in adults could prevent onset of asthma, especially in individuals with atopic diseases or a hereditary propensity to it. This could have major public health impact as asthma is among the most common chronic diseases in working-age adults. Prevention of common respiratory infections receives surprisingly little attention in current public health agendas. A question worth studying in the future is whether active treatment of respiratory infections could diminish development of asthma.

### Validity of results

In this study a high proportion of all new asthma cases in a geographically defined area were recruited by the health case system and with the help of the National Social Insurance Institution (response rate 86%). The response rate among controls was also relatively high (80%). Thus, any major selection bias is unlikely in this study population.

Asthma was defined on the basis of the occurrence of asthma symptoms and objective findings in extensive lung function measurements performed in accordance with the national guidelines (Table 1). This reduces misclassification of the outcome.

Information about the frequency and type of respiratory infections during the past 12 months and past 3 months was based on reporting in the questionnaire. Self-reported infections may include some misclassification. If both cases and controls tend to misreport their infections to some degree, this would lead to an underestimate of the real effect. If being diagnosed with asthma would influence the recall of respiratory infections so that these subjects would recall more infections, an information bias could take place leading to overestimation of the risk. However, it is unlikely that such bias would lead to so strong increases in risk that were observed in this study. In addition, this study aimed initially to investigate occupational exposures and asthma, so no special attention was paid to infections as potential determinants. Furthermore, the majority of the cases filled in the questionnaire before the diagnosis of asthma was made. We did not analyze specific infectious agents, but this would be interesting to study in the future.

In the multivariate analyses, all the relations were adjusted for a number of potential confounders in order to eliminate these factors as potential explanations for our results.

### Synthesis with previous knowledge

The role of respiratory infections in exacerbations of asthma has been established in both children and adults [6], [27]–[29], but the present study is the first one describing the role of acute respiratory infections in the onset of adult asthma. Our results are consistent with previous studies that have shown an increased risk of asthma onset in relation to respiratory infections in children [4], [6]. Lemanske et al. [7] and Jackson et al. [5] reported birth-cohort studies of children who were at high risk for asthma, and showed that wheezing episodes with a viral cause experienced during the first three years of age predicted a significant risk of asthma at six years of age. Another birth cohort study by Nafstad et al. [30] showed that respiratory infections in the first year of life were linked to increased risk of having bronchial obstruction at the age of two and asthma at the age of four. Consistently with present results in adults, the risk of asthma was related especially to lower respiratory infections.

### Conclusions

We present here new evidence that recently experienced respiratory tract infections are a strong determinant for the onset of adult asthma. A strongly increased risk of asthma was detected especially in relation to lower respiratory tract infections, but all types of respiratory infections studied, i.e. common cold, sinusitis, otitis, acute bronchitis, and pneumonia increased the risk of asthma onset. This could mean that reducing occurrence of respiratory infections might prevent onset of asthma in adulthood, especially in individuals with an atopic disease or hereditary propensity to it.
